# Energy Modeling of Neighbor Discovery in Bluetooth Low Energy Networks

**DOI:** 10.3390/s19224997

**Published:** 2019-11-16

**Authors:** Bingqing Luo, Jincheng Gao, Zhixin Sun

**Affiliations:** 1 Jiangsu Key Laboratory of Big Data Security & Intelligent Processing, Nanjing University of Posts and Telecommunications, Nanjing 210023, China; luobq@njupt.edu.cn (B.L.); gaojincheng98@126.com (J.G.); 2 Laboratory of Broadband Wireless Communication and Sensor Network Technology, Nanjing University of Posts and Telecommunications, Nanjing 210003, China

**Keywords:** Bluetooth low energy, neighbor discovery, energy consumption analysis, Internet of Things

## Abstract

Given that current Internet of Things (IoT) applications employ many different sensors to provide information, a large number of the Bluetooth low energy (BLE) devices will be developed for IoT systems. Developing low-power and low-cost BLE advertisers is one of most challenging tasks for supporting the neighbor discovery process (NDP) of such a large number of BLE devices. Since the parameter setting is essential to achieve the required performance for the NDP, an energy model of neighbor discovery in BLE networks can provide beneficial guidance when determining some significant parameter metrics, such as the advertising interval, scan interval, and scan window. In this paper, we propose a new analytical model to characterize the energy consumption using all possible parameter settings during the NDP in BLE networks. In this model, the energy consumption is derived based on the Chinese remainder theorem (CRT) for an advertising event and a scanning event during the BLE NDP. In addition, a real testbed is set up to measure the energy consumption. The measurement and experimental results reveal the relationship between the average energy consumption and the key parameters. On the basis of this model, beneficial guidelines for BLE network configuration are presented to help choose the proper parameters to optimize the power consumption for a given IoT application.

## 1. Introduction

Bluetooth low energy (BLE) is a personal LAN technology designed by Bluetooth special interest group (SIG). The specialty of BLE is as a short-range, cellular Internet of Things (IoT) wireless solution geared for a wireless world that demands ultra-low power usage [1].

As the number of the BLE devices increases, the performance of the neighbor discovery process (NDP) could have a significant impact on users’ experiences and devices’ life spans. It is validated that the parameters in the NDP, such as advertising interval, scan interval, and scan window, have a critical impact on two interdependent performance metrics: the discovery latency and the energy consumption. Both BLE 4.0 and BLE 5.0 provide a wide range of parameter options that support the requirements of IoT applications. To maximize the lifetime of the devices and meet the requirements of a given application, it is necessary to provide a method that can appropriately select parameters. As a result of its practical importance, academic efforts have been devoted to proposing analytical models that can optimize the parameter settings in the BLE NDP and obtain the best possible performance.

In [2,3], Cho et al. proposed a discovery latency and energy consumption model to analyze the performance of BLE neighbor discovery protocol. In contrast to other models, multiple advertisers were considered in the network. The simulation results showed that the parameters and the number of advertisers had a significant impact on the NDP performance. However, the analysis was given under the assumption of a continuous scanning scenario.

In [4], Liu et al. revealed, for the first time, the relationship between the discovery latency and the parameter settings in BLE neighbor discovery. The analyzed model was based on the effective scan window and was further enhanced in [5] to analyze the energy consumption during the neighbor discovery process. However, the model was constrained by the parameter range and only applied with the condition that the scan window was longer than advertising interval.

In [6], Philipp et al. proposed a sensitivity analytical model of different parameters on the energy consumption and evaluated the accuracy of the model using both discrete event simulations and actual measurements. In [7], Liendo et al. presented an extension of the model in [6] and a parameter optimization method to obtain the best parameter settings according to the application requirements.

In [8], a new analytical model was presented to characterize the performance of the BLE NDP with respect to the whole discovery time and energy consumption to provide the best parameter settings. Meanwhile, Liendo et al. proposed the neighbor discovery parameter optimization method to minimize the energy consumption for a wider range of IoT use cases in [9].

The above research was all based on probabilistic models, which, in some cases, limited the performance analysis. These probabilistic models could not capture the peaks in the advertising events, which exist in the measurements. Therefore, the probabilistic models’ results have accuracy problems.

An analytical model was suggested in [10] to obtain an upper bound for the discovery capacity. According to the measurements, additional scanning gaps that reduced the discovery capabilities were found in the scanning process. On the basis of the measurement and simulation results, the paper provided a guideline to select the desired parameter values.

Similar to [10], considering the scanning gaps, the real device behavior of BLE scannable undirected advertising events were experimentally modeled in [11], based on the new features of BLE 5.0 to characterize this new device discovery process. The discovery probability and discovery latency were fully analyzed under several advertising and scanning scenarios.

As described in [12], according to the Chinese remainder theorem (CRT), it was proved that two nodes would have some overlapping radio on-time within a bounded number of periods, even if they were running under the asynchronous neighbor discovery protocol.

The authors of [13] were the first to apply CRT to analyze the bounded latency of the BLE NDP for in-vehicle networks. The experiments in [13] showed that the BLE with coprime parameter values had a low discovery latency. However, there were no detailed energy performance analyses on the impact of parameter settings.

The most recent work using the CRT to model the NDP in [14] enhanced the analysis of the mean discovery latency and the energy consumption. However, the model is also based on the effective scan window, which only works when the advertising interval is smaller than the effective scan window.

Enlightened by the experimental models and CRT models in [12,14], this paper proposes an analytical energy model of the BLE NDP based on CRT to optimize the parameter settings for energy-sensitive IoT applications. The main contributions of this paper are listed as follows:

- Most previous works regarding the performance analysis of NDP used the probabilistic method. In this paper, we apply the Chinese reminder theory to model the neighbor discovery protocol in BLE networks, which is an effective way to solve the periodic interval problem;
- Unlike the previous work in [14], we introduce an energy consumption expression related to CRT for both an advertising event and a scanning event, and characterize the energy consumption covering all parameter ranges, including the case when the advertising interval is larger than the scan interval;
- Instead of using the effective scan window model proposed in previous works, we apply CRT to the distributed channels, without the constraint of the parameter settings;
- In order to validate the model, a real testbed was set up to measure the discovery latency and average current during the NDP.

The remainder of the paper is organized as follows. Section 2 briefly reviews the basic NDP (B-NDP) that is specified in [15] and the advanced NDP (A-NDP) in BLE 5.0 [16]. In Section 3, we propose an analytical energy model based on CRT and derive the average energy consumption for the NDP. The statistics results from the model and the measurement results are presented and discussed in Section 4. Finally, the paper concludes with Section 5.

## 2. Background

According to BLE 4.0 [1], the nodes in BLE networks have different roles based on their discovery states. Advertisers are the devices working in advertising mode, and scanners and initiators are the devices in scanning and initiating modes, respectively. In the advertising mode, the devices advertise packets in the advertising channels periodically, and then listen for responses from scanners with the channel order of 37-38-39. With the same channel order, the scanners periodically scan and wait for the advertising packets. Once the scanner receives the advertising information and is ready to connect with the advertisers, it turns into the initiator.

**Figure 1:**
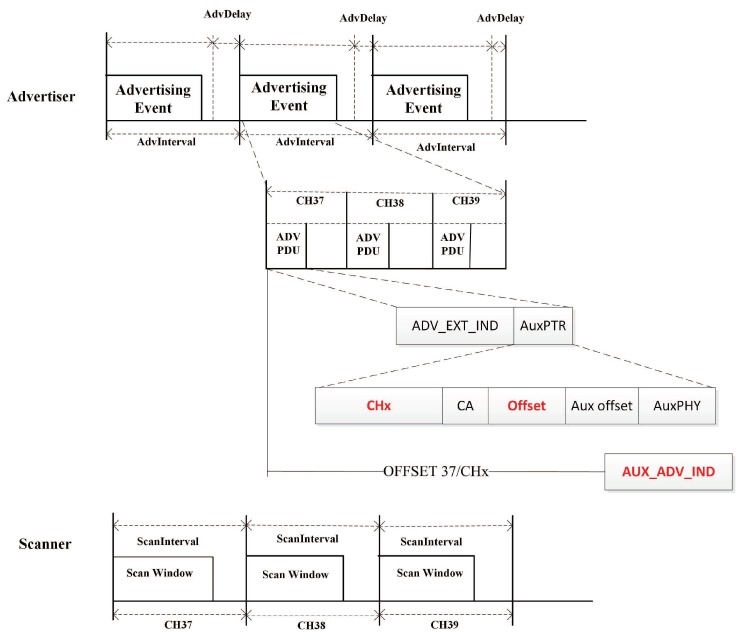
The process of Bluetooth low energy (BLE) neighbor discovery based on BLE 5.0.

Figure 1 shows the basic neighbor discovery process based on the BLE 5.0 [16]. Advertisers and scanners utilize the three primary channels (37-38-39) to send advertising packages and scan. Meanwhile, the key difference between the two versions is that during an AdvInterval period, each advertising packet Adv_PDU only contains ADV_EXT_IND and AuxPTR. These packages provide offset and channel messages instead of advertising data. Scanners will receive AUX_ADV_IND packages according to the offset on one specific data channel X (from Channel 0 to Channel 36). An AUX_ADV_IND package containing advertising data is a new package that is defined in BLE 5.0.

The BLE 5.0 specifies a wider range of feasible parameter values for the NDP than BLE 4.0, such as the AdvInterval, ScanWindow, and ScanInterval.

AdvInterval $T_{ADV}$ is one of the key parameters in neighbor discovery configuration, which determines the interval time between two consecutive advertising events. In an advertising event, the advertisers transmit the packets in the three predefined channels respectively. The time that the advertiser spends in each channel is denoted as $\tau_{wa}$. The fixed interval $\omega_{AI}$ and a pseudorandom delay μ are two parts of the AdvInterval.

ScanInterval $T_{SIN}$ denotes the period time that the scanning event happens in the three advertising channels. ScanWindow $\omega_{SW}$ is a fixed duration for scanners to scan and listen in the scanning state. Table 1 shows the list of the significant parameters for neighbor discovery that are specified in BLE 5.0, and these parameters have a wide range thus providing the BLE network with the abilities necessary to support a variety of applications.

As a result, the wide range of parameters cause problems in terms of finding the appropriate initial settings to meet the requirements of different BLE applications. The focus of our study is to analyze the impact of the parameters on the performance of neighbor discovery of BLE.

## 3. Modeling the Neighbor Discovery Process

In this section, we apply CRT to the BLE neighbor discovery process to model the energy consumption for advertising events and scanning events. We first present a study of asynchronous neighbor discovery based on CRT. Then, we discuss the energy consumption during the BLE NDP according to the measurement waveform. In what follows, we propose an analytical energy model to explore the relationship between energy consumption and parameter setting.

### 3.1. Asynchronous Neighbor Discovery and CRT

CRT [12] is a mathematical theorem that states that there exists an integer *x* satisfying the pair of simultaneous congruences for any two coprime numbers $n_{i}$ and $n_{j}$:

(1) $$\begin{array}{c} X\equiv m_{i}\left(modn_{i}\right),\\ X\equiv m_{j}\left(modn_{j}\right), \end{array}$$

where $m_{i}$ and $m_{j}$ are two integers. For example, the pair of simultaneous congruences x≡1(mod3) and x≡2(mod7) has the solution $x=16+21k,\;k\in Z^{+}$.

**Figure 2:**
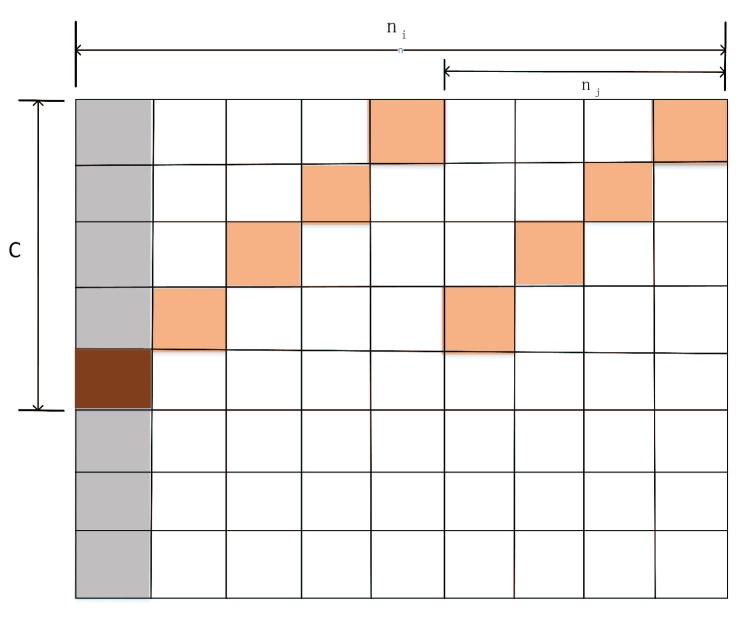
Discovery with asymmetric cycles ($n_{i}=9$, $n_{j}=4$).

According to CRT, we pick two numbers, $n_{i}$ and $n_{j}$, which denote the interval time that two nodes wake up from a sleeping state. Then, at the $\left(c-1\right)*n_{i}+1$ slot, the nodes discover each other if $n_{i}$ and $n_{j}$ are relatively prime, as illustrated in Figure 2. Therefore, at least one discovery is guaranteed at slot *x* within a period $n_{i}n_{j}$, regardless of the phase offsets of $m_{i}$ and $m_{j}$ due to their asynchronous clocks.

We can express *x* as

(2) $$X=x_{0}+kn_{i}n_{j},\;k\in Z^{+}.$$

When $x=x_{0}$, the nodes are turned on and can discover each other. Therefore, according to (2), $x_{0}$ denotes the first time when the two nodes meet. Furthermore, when $n_{i}$ and $n_{j}$ are coprime, the duty cycle can be expressed as

(3) $$DC=\frac{1}{n_{i}}\left(1+\frac{1}{c}\right)+\frac{1}{n_{j}}\left(1-\frac{1}{c}\right).$$

### 3.2. Energy Consumption in BLE NDP

**Figure 3:**
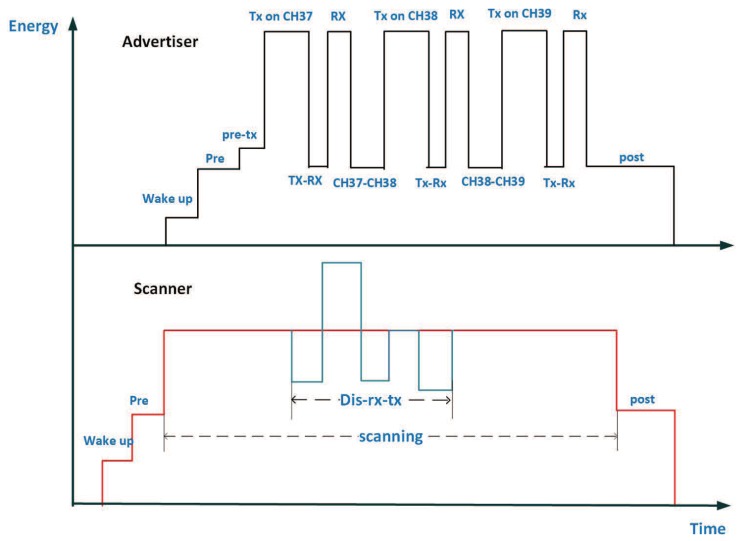
The energy consumption in the BLE neighbor discovery process (NDP).

In this section, we present the energy consumption waveform during an advertising event and a scanning event. As the measurement in [5,6], an energy consumption waveform can be presented as shown in Figure 3.

In order to establish the energy model during the NDP, Table 2 shows the operation and the energy constants of each state based on [6].

In the advertising event, the advertiser periodically broadcasts and listens on Channels (37, 38, and 39), which is reflected with the Tx, Rx, and Tx to Rx peaks in Figure 3. In addition, besides the advertising event, one advertising period includes the wake up state, transfer state, and some preparation states. Therefore, we can express the energy consumption with a period $E_{adv-p}$ as

(4) $$E_{adv-p}=E_{wake}+E_{pre}+E_{pre-tx}+3E_{tx}+3E_{rx}+3E_{tx-rx}+2E_{trans}+E_{post}.$$

Furthermore, the energy consumption on specified channel *n* with a period $E_{n}$ could be expressed as

(5) $$\begin{array}{c} E_{37}=E_{wake}+E_{pre}+E_{pre-tx}+E_{tx}+E_{rx}+E_{tx-rx}+E_{post}\\ E_{38}=E_{wake}+E_{pre}+E_{pre-tx}+2E_{tx}+2E_{rx}+2E_{tx-rx}+E_{trans}+E_{post}\\ E_{39}=E_{wake}+E_{pre}+E_{pre-tx}+3E_{tx}+3E_{rx}+3E_{tx-rx}+2E_{trans}+E_{post}. \end{array}$$

The energy consumption during a scanning event can be calculated as well as the advertising event. It should be pointed out that the scanning event contains two options: events with no reception and events with ADV_PDU packets.

Therefore, as shown in Figure 3, the charge that is consumed in a scanning period can be expressed as

(6) $$\begin{array}{cc} \; & E_{scan-no-re}=E_{wake}+E_{pre}+E_{scanning}+E_{post}\\ \; & E_{scan-re}=E_{wake}+E_{pre}+E_{pre-tx}+E_{scanning^{\prime }}+E_{dis-rx-tx}+E_{post}, \end{array}$$

where $E_{scanning^{\prime }}$ denotes the energy that is consumed during the active scanning and in the packet processing phase, $E_{scan-no-re}$ denotes the consumed energy during the scanning events with no reception, and $E_{scan-re}$ denotes the consumed energy during the scanning events with received ADV_PDU packets.

As discussed in the previous section, we have the energy consumption $E_{adv-n}$ for the advertiser on Channels (n=37,38,39):

(7) $$\begin{array}{cc} \; & E_{adv-37}=\left(c-1\right)*E_{adv-p}+E_{adv-37}\\ \; & E_{adv-38}=\left(c-1\right)*E_{adv-p}+E_{adv-38}\\ \; & E_{adv-39}=c*E_{adv-39}. \end{array}$$

In addition, the charge consumed by a scanner $E_{scan}$ is

(8) $$E_{scan}=E_{scan-re}+\left(c^{\prime }-1\right)*E_{scan-no-re},$$

where $c^{\prime }$ denotes the number of periods of the scanning events.

### 3.3. Analytical Energy Model of BLE NDP

In order to determine the relationship between the parameter settings and the energy consumption, in this section, we present an analytical energy model based on CRT, as an extension of the study in [17].

Instead of using the effective scan window to model the NDP, we proposed a distributed neighbor discovery analytical model for BLE networks, and applied CRT to three separated channels. On the basis of CRT, it could be ensured that there is at least a slot in which two nodes wake up together.

**Figure 4:**
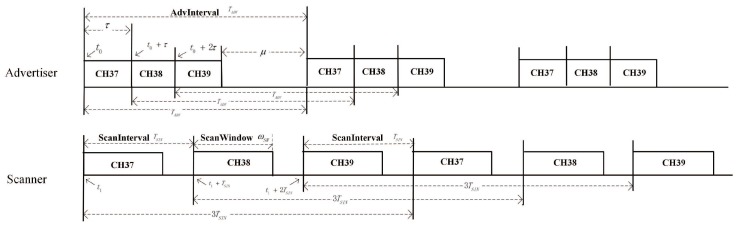
Distributed neighbor discovery analytical model in BLE.

To separate the three channels, the NDP in a cycle is separated into three components based on the channel number according to the same timeline. As shown in Figure 4, both the entering time and the periodic time of the advertising events and scanning events changed on the separated channels. If we assume that the advertiser entered the advertising mode at time $t_{0}$, and the scanner entered the scanning mode at time $t_{1}$, then, consistent with the advertising order, the advertising start time and the scanning start time for channel 38 is $t_{0}+\tau_{wa}$ and $t_{1}+T_{SIN}$, respectively, and those for channel 39 are $t_{0}+2\tau_{wa}$ and $t_{1}+2T_{SIN}$, respectively.

Another key parameter for CRT is the duty cycle. Different from the entering time, the advertiser and the scanner have the same duty cycle for all channels. As shown in Figure 4, the duty cycle of the advertiser could be expressed as $\frac{\tau_{wa}}{T_{ADV}}$, and for scanners, the duty cycle is $\frac{\omega_{SW}}{3T_{SIN}}$ ($T_{SCAN}=3T_{SIN}$).

Table 3 shows the key parameters for each channel based on CRT.

According to Table 3, we can develop the solution *x* for the CRT described by our model as

(9) $$\begin{array}{cc} \; & x_{on}^{37}=\Gamma \left(t_{0},t_{1},\;\frac{T_{ADV}}{\tau_{wa}},\;\frac{3T_{SIN}}{\omega_{SW}}\right)\\ \; & x_{on}^{38}=\Gamma \left(t_{0}+\tau_{wa},\;t_{1}+T_{SIN},\;\frac{T_{ADV}}{\tau_{wa}},\;\frac{3T_{SIN}}{\omega_{SW}}\right)\\ \; & x_{on}^{39}=\Gamma \left(t_{0}+2\tau_{wa},\;t_{1}+2T_{SIN},\frac{T_{ADV}}{\tau_{wa}},\frac{3T_{SIN}}{\omega_{SW}}\right). \end{array}$$

Thus, the smallest slot among all matching slots is $x_{0}=min\left(x_{on}^{37},\;x_{on}^{38},x_{on}^{39}\right)$. Let $\theta (t_{0},t_{1})$ denote the interval from the initiation time of the advertiser to $x_{0}$. Let $\phi (t_{0},t_{1})$ denote the interval from the initiation time of the scanner to $x_{0}$. Then,

(10) $$\begin{array}{c} \theta \left(t_{0},t_{1}\right)=x_{0}-t_{0}\\ \phi \left(t_{0},t_{1}\right)=x_{0}-t_{1}. \end{array}$$

Therefore, as defined in Figure 2, it is noticed that *c* and $c^{\prime }$ can be expressed as

(11) $$\begin{array}{c} c=\frac{\theta \left(t_{0},t_{1}\right)*\tau_{wa}}{T_{ADV}}\\ c^{\prime }=\frac{\phi \left(t_{0},t_{1}\right)*\omega_{SW}}{3T_{SIN}}. \end{array}$$

Meanwhile, we can calculate the energy consumption $E_{adv}^{x}$ when the advertising events stop in the *c*th period on the channels as

(12) $$\begin{array}{cc} \; & E_{adv}^{37}=\left(\frac{\theta \left(t_{0},t_{1}\right)*\omega_{wa}}{T_{ADV}}-1\right)*E_{adv-p}+E_{adv-37}\\ \; & E_{adv}^{38}=\left(\frac{\theta \left(t_{0},t_{1}\right)*\omega_{wa}}{T_{ADV}}-1\right)*E_{adv-p}+E_{adv-38}\\ \; & E_{adv}^{39}=\frac{\theta \left(t_{0},t_{1}\right)*\omega_{wa}}{T_{ADV}}*E_{adv-39}. \end{array}$$

To simplify the equation, the energy consumption for advertisers $E_{adv}^{x}$ can be expressed as

(13) $$\begin{array}{c} E_{adv}^{x}=\left(\frac{\theta \left(t_{0},t_{1}\right)*\omega_{wa}}{T_{ADV}}-1\right)*E_{adv-p}+E_{adv-x}\\ x=37,38,39 \end{array}.$$

In addition, the same as advertising events, the scanner energy consumption $E_{scan}$ could be given as

(14) $$E_{scan}=E_{scan-re}+\frac{\phi \left(t_{0},t_{1}\right)*\omega_{SW}}{3T_{SIN}}-1*E_{scan-no-re}.$$

Finally, we assume that the advertiser and scanner initially start at any slot within $\left[0,T_{ADV}\right]$ and $\left[0,3T_{SIN}\right]$, respectively, independently and with the same probability. As a result, the average energy consumption for advertisers and scanners during the BLE NDP is

(15) $$\begin{array}{cc} \; & \bar{E_{adv}}=\frac{1}{T_{ADV}}\sum_{t_{0}=0}^{T_{ADV}}E_{adv}^{x},\;x=37,38,39\\ \; & \bar{E_{scan}}=\frac{1}{3T_{SIN}}\sum_{t_{1}=0}^{3T_{SIN}}E_{scan}. \end{array}$$

## 4. Experimental Results

**Figure 5:**
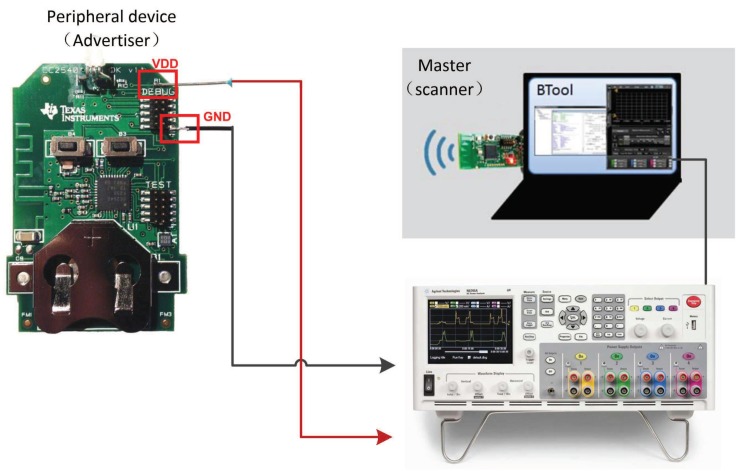
The architecture of the testbed.

In this section, to validate the analytical models, we have developed a BLE energy measurement program, which fully complies with the BLE specification 4.2, on a real testbed using the Texas Instruments SimpleLink Bluetooth low energy CC2540dk-mini kit with the Software Development Kit BLE-Stack [18]. The included key fob board operates as a BLE peripheral device, and contains modifiable software that can be tailored towards different parameters. A CC2540 USB Dongle acting as a master connects to a PC’s USB port. Using BTool (Windows PC application) along with the included CC2540 USB Dongle, the BLE stack can be tested and verified while developing the custom test program. When the devices being tested are set up properly, a few simple hardware modifications are required to implement the current measurement. The detailed implementation steps can be found in [19]. The architecture of the testbed is shown in Figure 5.

During the measurement experiments, we use the USB Dongle and the BTool to capture the BLE packets, and utilize a DC power analyzer to measure the current and record the time of advertising events during the discovery process [20]. Since the average current is the value that is highly dependent on the parameter settings, the average power consumption can be calculated from the average current consumption for the advertising and the discover latency. In the experiments, the central device starts at a random time to discover the advertiser. The typical experimental procedure is to fix two parameters and to vary the other one. The measurements for every parameter setting is repeated 30 times to calculate the average current.

In the experiments, the starting times of the scanner and the advertiser are randomly chosen from the intervals $\left[0,T_{ADV}\right]$ and $\left[0,3T_{SIN}\right]$. The other default parameter configurations are given according to [5,6] in Table 4.

**Figure 6:**
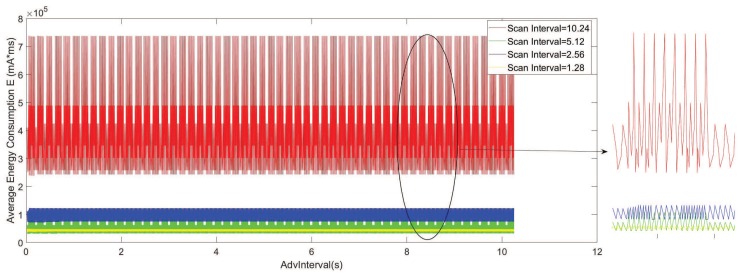
Average energy consumption of the advertisers according to AdvInterval. ($T_{ADV}\in$ [0.02 s, 10.24 s], $T_{SIN}$ = 1.28 s, 2.56 s, 5.12 s, 10.24 s, $\omega_{SW}=1.28$ s).

Figure 6 shows the modeled statistics of the average energy consumption for the advertiser during the NDP, with AdvInterval ranging from 0.02 to 10.24 s; ScanInterval $T_{SIN}$ = 1.28 s, 2.56 s, 5.12 s, 10.24 s; and ScanWindow $\omega_{SW}$ = 1.28 s.

**Figure 7:**
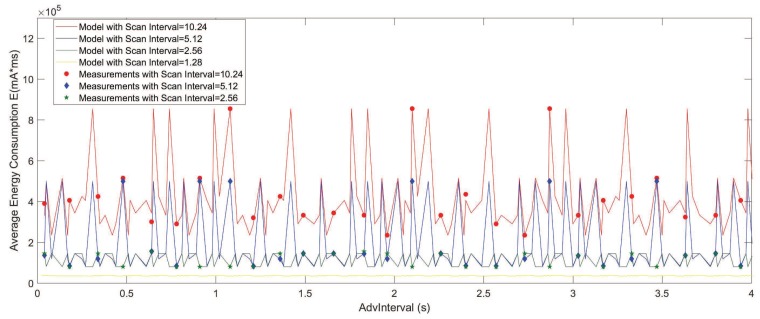
Average energy consumption results of the model and the measurements. ($T_{ADV}\in$ [0.02 s, 4 s], $T_{SIN}$ = 1.28 s, 2.56 s, 5.12 s, 10.24 s, $\omega_{SW}$ = 1.28 s).

Figure 7 shows the detailed results of both the measured and modeled statistics with AdvInterval ranging from 0.02 to 4 s. The measurement results show the same pattern as the results that were obtained from the model.

Figure 6 and Figure 7 reveal some interesting results. Contrary to the intuition that more frequent advertising leads to larger energy consumption, periodic high energy consumption could be observed in the analysis results and the measurements. In addition, increasing the value of the AdvInterval does not increase the energy if peaks are avoided. However, a higher ScanInterval leads to a larger range of maximums and minimums of the peaks. Using CRT, it can be shown that the largest solution is for a pair of advertisers and scanners in a duty cycle, which happens periodically in every $n_{i}n_{j}$. Furthermore, in this case when $\omega_{SW}\ll T_{SIN}$, there is a possibility that the scanner missed the advertiser during the specific scan period, and the energy consumption of the idle advertising when $T_{idle}=T_{SIN}-\omega_{SW}$ increases significantly, as showed in Figure 7 when ScanWindow $\omega_{SW}$ = 1.28 s and ScanInterval $T_{SIN}$ = 10.24 s.

Consequently, the peaks show a very large average energy consumption, which should be avoided during the configuration. Similar results were observed in the previous discovery latency analysis works of [6,7,17]. In addition, it is noted that the larger ScanInterval can lead to a larger range between the maximum value and the minimum value of the average energy consumption with a fixed ScanWindow. As a result, one should be more cautious and choose the AdvInterval accordingly if the requirement needs a large ScanInterval.

**Figure 8:**
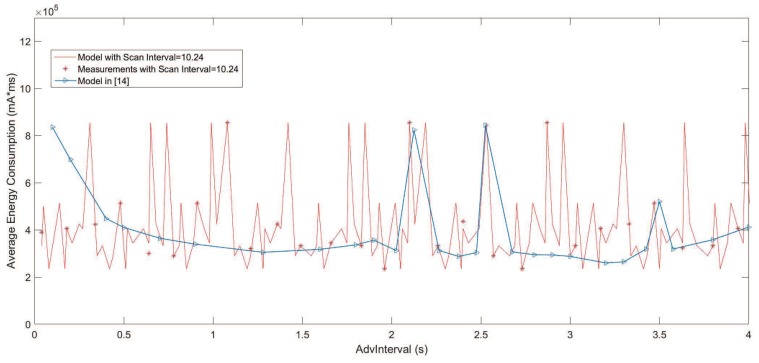
Average energy consumption results of the models.

Figure 8 shows the results compared with the model in [14] when ScanInterval $T_{SIN}$ = 10.24 s and ScanWindow $\omega_{SW}$ = 1.28 s. It is obvious that the results from the model proposed in this paper has a higher accuracy than the model in [14], especially when $T_{ADV}\geq \omega_{SW}$. This is because the model in [14] was based on the effective ScanWindow $d_{e}$. In model [14], the effective ScanWindow $d_{e}=\omega_{SW}-d_{a}$, and $d_{a}$ denotes the advertising time on one channel during an advertising event. When $d_{a}\geq \omega_{SW}$, it leads to the failure of the analysis in the model based on the effective ScanWindow.

**Figure 9:**
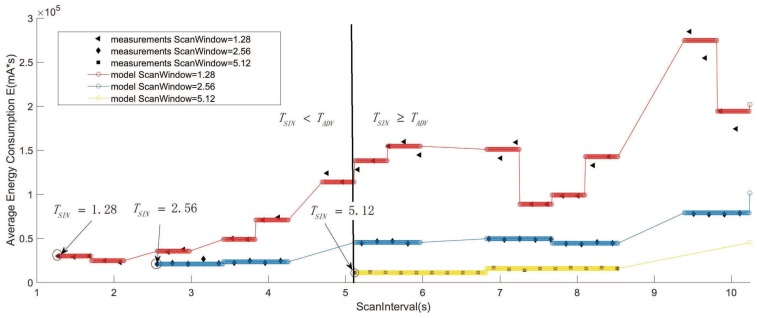
Average energy consumption of scanners according to the scanInterval ($T_{ADV}$ = 5.12 s, $T_{SIN}\in$ [1.28 s, 10.24 s], $\omega_{SW}$ = 1.28 s, 2.56 s, 5.12 s).

Figure 9 shows the expected energy consumption of the advertiser during a discovery process by varying the ScanInterval for different ScanWindows and with a fixed AdvInterval of $T_{ADV}$ = 5.12 s. As can be seen, the results from the measurement and results that were obtained from the model are close. The curves show that for each ScanWindow, the energy consumption steadily increases when the ScanInterval increases from 0.02 to 10.24 s. Meanwhile, from the comparison of the three situations, when $T_{ADV}=\omega_{SW}$, it is easy to achieve a low energy consumption for advertisers. Furthermore, for larger ScanWindows, the ScanInterval has less impact on the energy consumption of advertisers. The reason is that the larger ScanWindow reduces the energy of idle advertising events.

**Figure 10:**
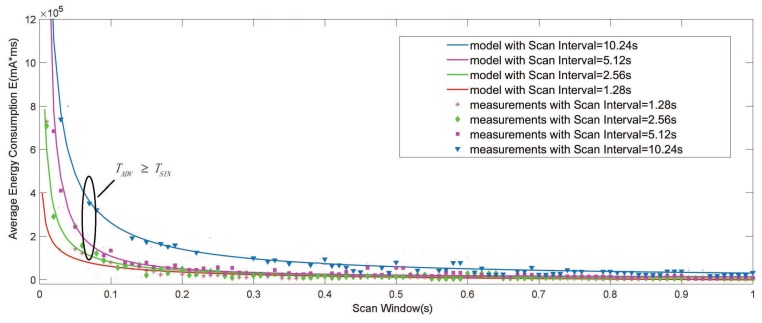
Average energy consumption with respect to the ScanWindow. ($T_{ADV}$ = 5.12 s, $T_{SIN}$ = 1.28 s, 2.56 s, 5.12 s, 10.24 s, $\omega_{SW}\in \left[0,T_{SIN}\right]$).

Figure 10 illustrates the results of the energy consumption versus varied ScanWindows ranging from 0 to $T_{SIN}$ for different ScanIntervals and a fixed AdvInterval of $T_{ADV}$ = 5.12 s. We compared the results from the model to the measurements, and they are also close.

The figures clearly show that as the ScanWindow increases, the energy consumption has a steep decrease when $\omega_{SW}\leq$ 0.2 s. This can be explained by the fact that the ScanWindow mainly affects the chance for advertisers and scanners to cross on one of the advertising channels. When the ScanWindow is large enough, the ScanWindow has a simple impact on the energy consumption. However, when the ScanWindow is less than one specific value, the larger ScanInterval will require more energy from the advertisers due to the higher frequency of idle advertising.

In summary, the experimental results validate the model and characterize the energy consumption for the BLE parameters. In the meantime, from the experimental results, it can be stated that there is a principle to properly set the parameters to meet the requirements of a given IoT application. For devices with frequent advertising events, a good trade-off is to set $T_{ADV}\geq T_{SIN}$, and if a large scanInterval is needed, we suggest using continuous scanning to achieve the fastest possible discovery and the smallest possible energy consumption. For devices that are expected to achieve the longest life spans with longer idle-advertising, a good choice is to set $T_{ADV}<T_{SIN}$ and possibly a lower ScanInterval.

## 5. Conclusions

We proposed an analytical energy model to characterize the energy consumption during the NDP in BLE networks. In this model, the energy consumption for an advertising event and a scanning event is calculated based on CRT. The model is validated using real testbed measurements, and we show that the average energy consumption varies with all possible standard parameter settings. Accordingly, on the basis of the given requirements of the IoT applications, parameter setting guidance could be derived using the model.

## Figures and Tables

**Table 1:** BLE significant parameters and recommended values.

Item	Notation	Value
Fixed Interval	$\omega_{AI}$	20 ms $\leq \omega_{AI}\leq$ 10,485.759375 s
AdvDelay	μ	$0\leq \mu \leq \mu_{MAX}\leq$ 10 ms
AdvInterval	$T_{ADV}$	$\omega_{AI}+\mu$
Advertising period per channel	$\tau_{wa}$	$0\leq \tau_{wa}\leq$ 10 ms
ScanWindow	$\omega_{SW}$	$0\leq \omega_{SW}\leq T_{SIN}$
ScanInterval	$T_{SIN}$	$0\leq T_{SIN}\leq$ 10.24 s

**Table 2:** The operation and the energy constants of each state.

State	Operation	Constants
wake up	the device wakes up from sleeping	$E_{wake}$
pre	the BLE protocol stack prepares for sending and listening	$E_{pre}$
pre-tx	the device turns on in preparation for advertising	$E_{pre-tx}$
Tx	the device transmits an advertising packet	$E_{Tx}$
Rx	the device listens for a packet	$E_{Rx}$
Tx-Rx	the state transfers from Tx to Rx	$E_{Tx-Rx}$
CH37-CH38	the advertising event on Channel 37 transfers to Channel 38	$E_{inter-ch}$
CH38-CH39	the advertising event on Channel 38 transfers to Channel 39	$E_{inter-ch}$
post	the BLE protocol stack prepares for the next advertising event	$E_{post}$
scanning	the device listens for an advertising packet	$E_{scanning}$
Dis-rx-tx	the states when the device receives an advertising packet	$E_{dis-rx-tx}$

**Table 3:** Key parameters of the distributed neighbor discovery model.

Item	CH37	CH38	CH39
$\frac{1}{n_{i}}$ (advertiser duty cycle)	$\frac{\tau_{wa}}{T_{ADV}}$	$\frac{\tau_{wa}}{T_{ADV}}$	$\frac{\tau_{wa}}{T_{ADV}}$
$\frac{1}{n_{j}}$ (scan duty cycle)	$\frac{\omega_{SW}}{3T_{SIN}}$	$\frac{\omega_{SW}}{3T_{SIN}}$	$\frac{\omega_{SW}}{3T_{SIN}}$
$m_{i}$ (advertiser phase offset)	$t_{0}$	$t_{0}+\tau_{wa}$	$t_{0}+2\tau_{wa}$
$m_{j}$ (scanner phase offset)	$t_{1}$	$t_{1}+T_{SIN}$	$t_{1}+2T_{SIN}$

**Table 4:** The default parameters set in the measurements.

Parameters	Value	Parameters	Value
$\tau_{wa}$	7.46 ms	$E_{wake}$	2.40 mA·ms
$E_{pre}$	4.40 mA·ms	$E_{pre-tx}$	2.00 mA·ms
$E_{Tx}$	6.55 mA·ms	$E_{Tx-Rx}$	0.77 mA·ms
$E_{Rx}$	2.01 mA·ms	$E_{inter-ch}$	1.11 mA·ms
$E_{post}$	7.03 mA·ms	$E_{dis-rx-tx}$	6.84 mA·ms
$E_{scanning}$	11.33 mA·ms		
